# Half-Life of African Swine Fever Virus in Shipped Feed

**DOI:** 10.3201/eid2512.191002

**Published:** 2019-12

**Authors:** Ana M.M. Stoian, Jeff Zimmerman, Ju Ji, Trevor J. Hefley, Scott Dee, Diego G. Diel, Raymond R.R. Rowland, Megan C. Niederwerder

**Affiliations:** Kansas State University, Manhattan, Kansas, USA (A.M.M. Stoian, T.J. Hefley, R.R.R. Rowland, M.C. Niederwerder);; Iowa State University, Ames, Iowa, USA (J. Zimmerman, J. Ji);; Pipestone Applied Research, Pipestone, Minnesota, USA (S. Dee);; Cornell University, Ithaca, New York, USA (D.G. Diel)

**Keywords:** African swine fever virus, ASFV, feed ingredients, half-life, transoceanic shipping, trans-Atlantic shipping model, viruses, virus stability, virus persistence

## Abstract

African swine fever virus is transmissible through animal consumption of contaminated feed. To determine virus survival during transoceanic shipping, we calculated the half-life of the virus in 9 feed ingredients exposed to 30-day shipment conditions. Half-lives ranged from 9.6 to 14.2 days, indicating that the feed matrix environment promotes virus stability.

African swine fever virus (ASFV) is the most significant threat to pork production worldwide. Over the past year, the virus has emerged in new countries and continents, including Belgium (*1*), and has rapidly disseminated throughout China and several other countries in Asia (*2**,**3*). Without effective vaccines or treatment, infection with ASFV results in severe disease in swine, high mortality rates, and preventive culling to halt virus spread. Since the 2013 introduction of porcine epidemic diarrhea virus in the United States, feed and feed ingredients have been recognized as potential routes for transboundary spread of swine diseases (*4*). Recent work has demonstrated that the stability of ASFV strain Georgia 2007 across animal feed ingredients is broad and that the virus survives in ingredients subjected to environmental conditions mimicking trans-Atlantic shipment (*5*). Furthermore, experimental infection with ASFV Georgia 2007 can occur through the natural consumption of contaminated plant-based feed; the likelihood of infection increases after repeated consumption of a batch of feed (*6*). Field reports have also implicated contaminated feed as playing a role in the introduction and transmission of ASFV on farms in China and Latvia (*7**–**9*).

We previously evaluated the stability of ASFV in various feed ingredients during a simulated 30-day trans-Atlantic voyage. We used those data to prepare rough estimates for the half-life of ASFV in each ingredient (*5**,**10*). However, half-life calculations were based on the limited data available at the time, including 2 time points representing inoculation dose and titers at the conclusion of the study and insufficient replicates from which to calculate SEs or 95% CIs around the half-life estimates. For this study, our objective was to improve the accuracy of ASFV half-life estimates by increasing the number of time points and replicates in the same trans-Atlantic model.

## The Study

We used 9 feeds or feed ingredients for this study. We programmed an environmental chamber with the environmental conditions of humidity and temperature, which fluctuated every 6 hours, over a 30-day simulated trans-Atlantic shipment (*11*). We added 5 g of each gamma-irradiated feed ingredient to 50-mL conical tubes before inoculating them with 100 µL of 10^5^ 50% tissue culture infective dose (TCID_50_) of ASFV. We used ASFV Georgia 2007/1 (*12*) because of its similarity to currently circulating isolates (*3*). Negative controls consisted of complete feed samples in meal form with 100 µL of sterile phosphate-buffered saline (PBS) added. Positive controls consisted of 5 mL of RPMI 1640 medium (Gibco, https://www.thermofisher.com) lacking feed with 100 µL of 10^5^ TCID_50_ ASFV. After addition of virus or PBS, we vortexed samples for 10 s and covered each tube with a vented cap for incubation. After removing the samples from the environmental chamber, we added 15 mL of sterile PBS and replaced the vented caps with solid caps. We organized samples in duplicate into 4 replicate batches representing 4 time points and simulated the trans-Atlantic shipping model over 2 separate 30-day periods. We used 144 titrations for the half-life calculations in feed (4 time points × 4 replicates = 16 titers/feed ingredient) and duplicate titers over 4 time points to calculate half-life in RPMI medium. We tested samples for ASFV on days 1, 8, 17, and 30 after contamination. The first sample was collected at 1 day after contamination to allow the virus to stabilize within each matrix.

ASFV was quantified by virus titration as described previously (*5*). We vortexed samples for 10 s and then centrifuged at 10,000 × *g* for 5 min at 4°C. Supernatant from each sample was stored at −80°C. We collected porcine alveolar macrophages for virus isolation by lung lavage of 3–5-week-old pigs and cultured for 1 day in RPMI medium supplemented with 10% fetal bovine serum and antibiotics in a 37°C 5% CO_2_ incubator. We prepared 2-fold serial dilutions in RPMI medium in triplicate, added dilutions to monolayers of porcine alveolar macrophages in 96-well plates, and incubated for 1 h at 37°C. Cells were washed again and RPMI medium replaced. After 4 days at 37°C, the cells were fixed with 80% acetone for 10 min and stained with mouse anti-p30 primary monoclonal antibody (1:6,000 dilution). We incubated plates at 37°C for 1 h and washed 3 times with PBS before addition of goat anti-mouse Alexa Fluor 488 secondary antibody (Invitrogen, https://www.thermofisher.com; 1:400 dilution), followed by 1-h incubation at 37°C. We viewed cells under a fluorescence microscope and calculated the log_10_ TCID_50_/mL according to the Spearman-Karber method (*13*).

For all sample types, we calculated the half-life and corresponding 95% CI. The half-life analysis was performed by fitting a linear regression model to the data by using R version 3.5.2 (https://www.r-project.org), with the natural log of the virus endpoint titers as the response variables and time as the explanatory variables. We estimated the slope and SE of the respective lines by using these regression models and half-lives calculated as −log_e_(2)/slope as previously described (*14*). We calculated the SE for each half-life by multiplying the SE of the slope by log(2) divided by the square of the slope. We calculated the upper and lower bounds of the 95% CI as the estimated half-life plus/minus the product of the SE times the critical value of a *t* distribution with quantile as 0.025 and degrees of freedom as n – 2, where n is the sample size for that ingredient (*14*).

Environmental conditions during the course of the trans-Atlantic model (Figure, panel A) were a mean + SD temperature of 12.3 ± 4.7°C (range 0–26°C) and a mean + SD humidity of 74.1% ± 19.2% (range 20%–100%). Negative control samples remained negative. All ASFV-inoculated samples showed detectable quantities of infectious ASFV (Figure, panel B). The half-life estimate in the RPMI-positive control was shorter than that for all feed ingredients tested: 8.3 + 0.3 days (95% CI 7.7–9.0 days) (Table). The virus half-life was longest in complete feed: 14.2 + 0.8 days (95% CI 12.4–15.9 days). Of note, for conventional versus organic soybean meal, the half-life of ASFV differed by >3 days: 9.6 + 0.4 days (conventional soybean meal) and 12.9 + 0.6 days (organic soybean meal). The relative stability in feed may be the result of variable protein, fat, or moisture content among ingredients. Overall, the mean half-life for ASFV in all animal feed ingredients was 12.2 days.

**Figure:**
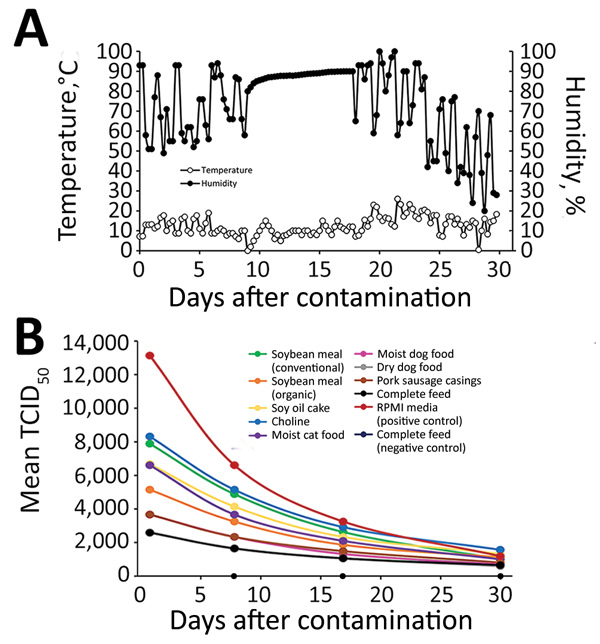
Decay of African swine fever virus (ASFV) Georgia 2007 in feed ingredients exposed to temperature and humidity conditions simulating a 30-day trans-Atlantic shipment. A) Temperature and humidity conditions, which fluctuated every 6 hours during the course of the 30-day environmental model. Environmental conditions were based on the availability of historical data logged from April 5, 2011, through May 4, 2011 (*5**,**11*) to model trans-Atlantic shipment from Warsaw, Poland, to Des Moines, Iowa, USA. B) Mean TCID_50_ of ASFV Georgia 2007 quantified on porcine alveolar macrophages at 1, 8, 17, and 30 days after contamination for different types of feed and controls. Feed ingredients were inoculated with 10^5^ TCID_50_ ASFV based on previous half-life calculations (*5**,**10*) and the infectious dose in feed (*6*). TCID_50_, 50% tissue culture infective dose.

**Table:** Half-life of African swine fever virus Georgia 2007 in animal feed ingredients subjected to temperature and humidity conditions simulating a 30-d transoceanic shipment*

Feed or feed ingredient	Mean titer on day 30†	Half-life ± SE	95% CI for half-life estimates	Previous titer on day 30 (*5**,**10*)†	Previous half-life estimates (*5**,**10*)
Soybean meal (conventional)	10^3.0^	9.6 ± 0.4	8.7–10.4	10^3.0^	4.6
Soybean meal (organic)	10^3.0^	12.9 ± 0.6	11.5–14.3	10^3.1^	4.7
Soy oil cake	10^3.1^	12.4 ± 0.9	10.4–14.3	10^3.2^	5.0
Choline	10^3.2^	11.9 ± 0.5	10.9–12.9	10^3.2^	5.1
Moist cat food	10^3.0^	10.6 ± 0.5	9.5–11.7	10^3.0^	4.6
Moist dog food	10^2.8^	11.7 ± 0.4	10.8–12.6	10^2.8^	4.2
Dry dog food	10^2.7^	13.1 ± 0.4	12.3–14.0	10^2.8^	4.1
Pork sausage casings	10^2.9^	13.1 ± 0.7	11.6–14.6	10^2.9^	4.4
Complete feed	10^2.7^	14.2 ± 0.8	12.4–15.9	10^2.9^	4.3
RPMI medium	Not determined	8.3 ± 0.3	7.7–9.0	10^3.0^	4.7

*Values listed in days unless otherwise indicated. Feed ingredient selection based on use in swine feed or volume of ingredient imported into the United States from China each year (*5*). Samples subjected to temperature (mean 12.3°C) and relative humidity (mean 74.1%) conditions in an environmental chamber programed to simulate transoceanic shipment. Complete feed samples were in meal form. †Mean titer of duplicate samples listed as 50% tissue culture infective dose.

## Conclusions

Although the high stability of ASFV in contaminated pork products and blood has been appreciated for decades (*15*), the stability of ASFV in plant-based feed has been recognized only recently (*5*). Our previous estimation of the half-life of ASFV in feed ingredients was based on the limited data we had available, including inoculation dose and 18 titers quantified at 1 time point during the 30-day model (*5**,**10*). In this study, we quantified viral decay at several time points over the 30-day model and increased sample size, which enabled us to calculate SEs and 95% CIs around the half-life estimates. In general, this updated modeling approach resulted in longer half-life estimates across all matrices.

This study provides quantitative data on the half-life of ASFV Georgia 2007 in animal feed ingredients exposed to moderate temperature and humidity conditions simulating transoceanic shipment. The longer virus half-lives in feed compared with half-lives in media support the concept that the feed matrix provides an environment that increases ASFV stability. Furthermore, these data provide additional evidence supporting the ability of plant-based feed ingredients to promote survival of ASFV should these products become contaminated.
